# Supplementary material for: CTCF couples long-range loop extrusion and diffusion to mediate a diverse Igκ repertoire
Source: Nat Commun. 2025 Dec 11;17:751. doi: 10.1038/s41467-025-67438-5 (PMC12820099; doi:10.1038/s41467-025-67438-5)
Supplement: Supplementary file 1 — Supplementary Information [file 41467_2025_67438_MOESM1_ESM.pdf]

Supplementary Information

CTCF couples long-range loop extrusion and diffusion to mediate a diverse *Igκ* repertoire

Emma L. Bush[1,#], Brigette Berke-Reynolds[1,#], Kaitlyn M. Hutchins[1], Xinrui Yu[2], Jorge A. Colón-Rosado[3], Fujung Chang[1], John Curran[1], Jiaxin Yang[2], Liangliang Sun[3], Jianrong Wang[2*] & Yu Zhang[1*]

[1]Department of Microbiology, Genetics and Immunology, Michigan State University, East Lansing, MI 48824, USA

[2]Department of Computational Mathematics, Science and Engineering, Michigan State University, East Lansing, MI 48824, USA

[3]Department of Chemistry, Michigan State University, East Lansing, MI 48824, USA

[#]These authors contributed equally

[*]Correspondence and requests for materials should be addressed to: wangj164@msu.edu (J.W.) and yzhang22@msu.edu (Y.Z.)

Section A: Supplementary Figures
- Supplementary Figures 1-10

Section B: Supplementary Tables
- Supplementary Tables 1-2

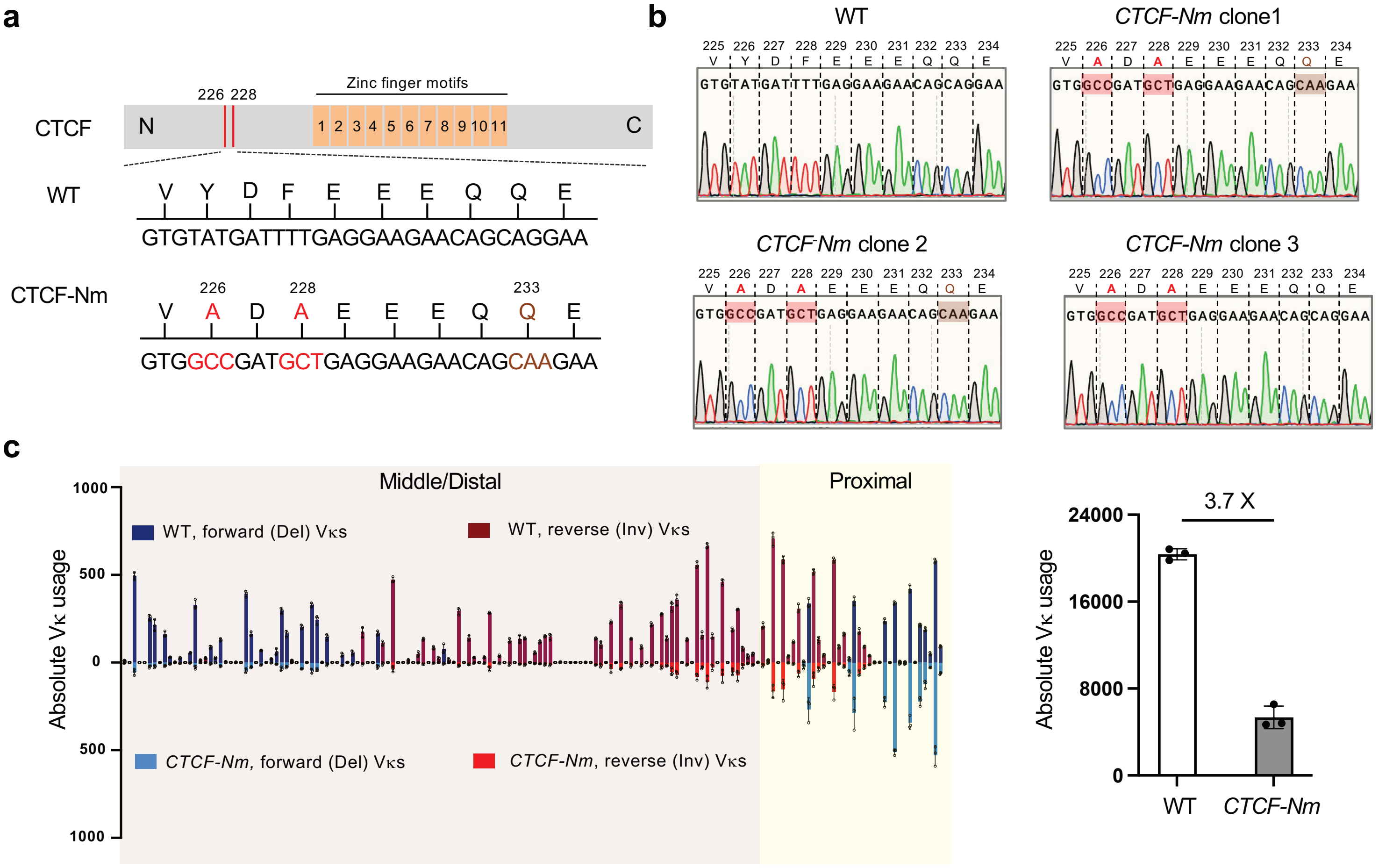

Supplementary Figure 1. Characterization of the *CTCF-Nm* line. (**a**) Diagram of the CTCF(Y226A/F228A) mutation. The template ssODN includes an additional silent mutation at Q233 to disrupt the PAM motif of CRISPR/Cas9 following the replacement. (**b**) Sanger sequencing confirmation of three biologically independent CTCF mutant clones. All of them contain the CTCF(Y226A/F228A) mutation, and two clones also contain the Q233 silent mutation. (**c**) Left, HTGTS-V(D)J-Seq analysis of absolute count of individual Vκ usage in WT (top) and *CTCF-Nm* (bottom) cells. Libraries were normalized to 252,701 total aligned reads (raw junctions + germline reads). Right, Comparison of the absolute count of total Vκ usage in WT and *CTCF-Nm* cells. Data represents mean ± SD from three independent replicates.

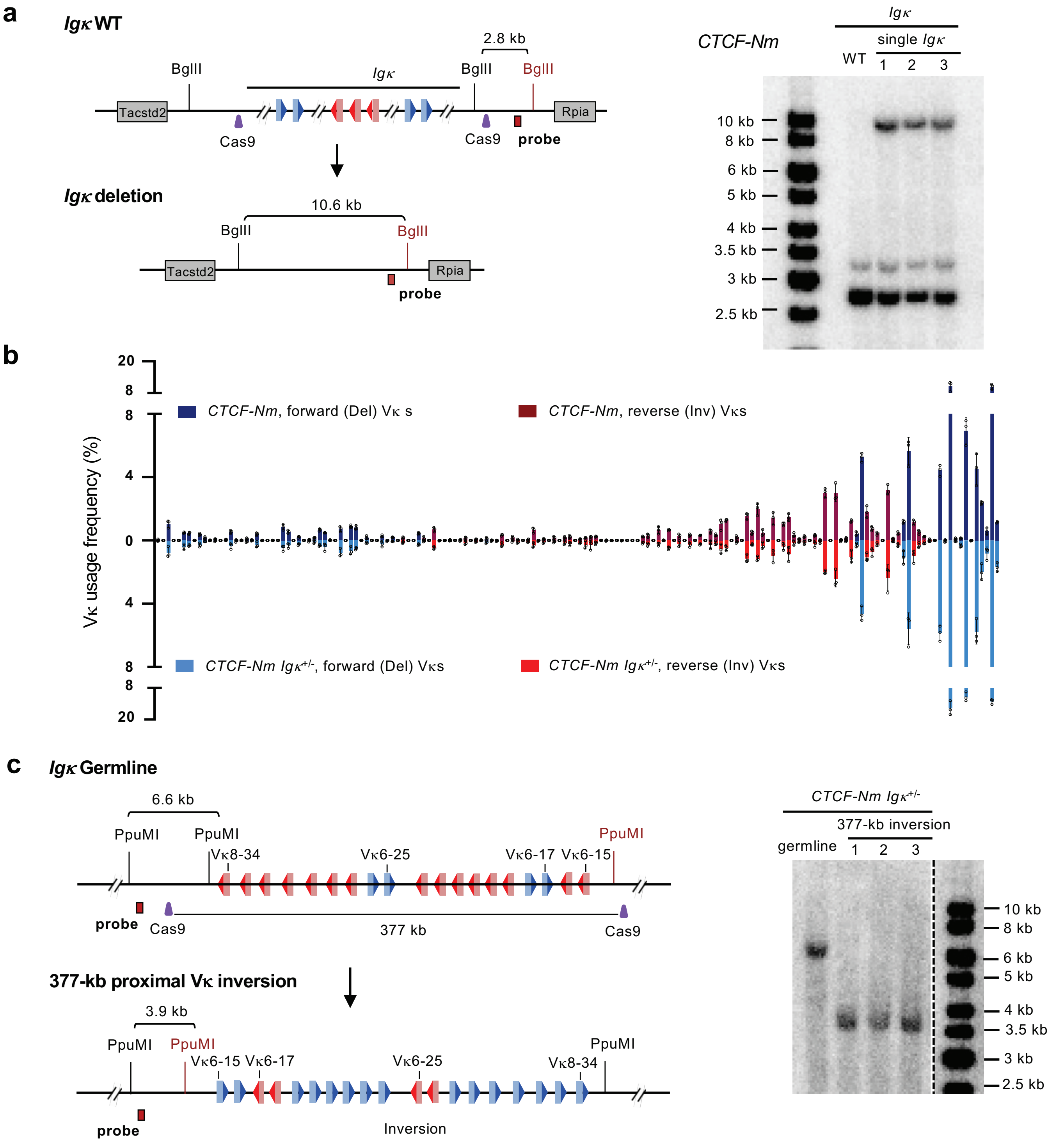

Supplementary Figure 2. Characterization of the *CTCF-Nm Igκ*$^{+/-}$ and *CTCF-Nm Igκ*$^{377\text{-}inv}$ lines. (**a**) Left, schematic illustrating the Southern blot strategy to verify deletion of a copy of *Igκ* allele. Right, southern blot confirmation of three biologically independent *Igκ*$^{+/-}$ clones derived from the *CTCF-Nm* line. (**b**) HTGTS-V(D)J-Seq analysis of Vκ utilization frequency in *CTCF-Nm* (top) and *CTCF-Nm Igκ*$^{+/-}$ (bottom) lines. Data represents mean ± SD from three independent replicates. (**c**) Left, Schematic illustrating the Southern blot strategy to verify inversion of the 377-kb proximal Vκ region. Right, Southern blot confirmation of three independent *Igκ*$^{377\text{-}inv}$ clones derived from the *CTCF-Nm Igκ*$^{+/-}$ line.

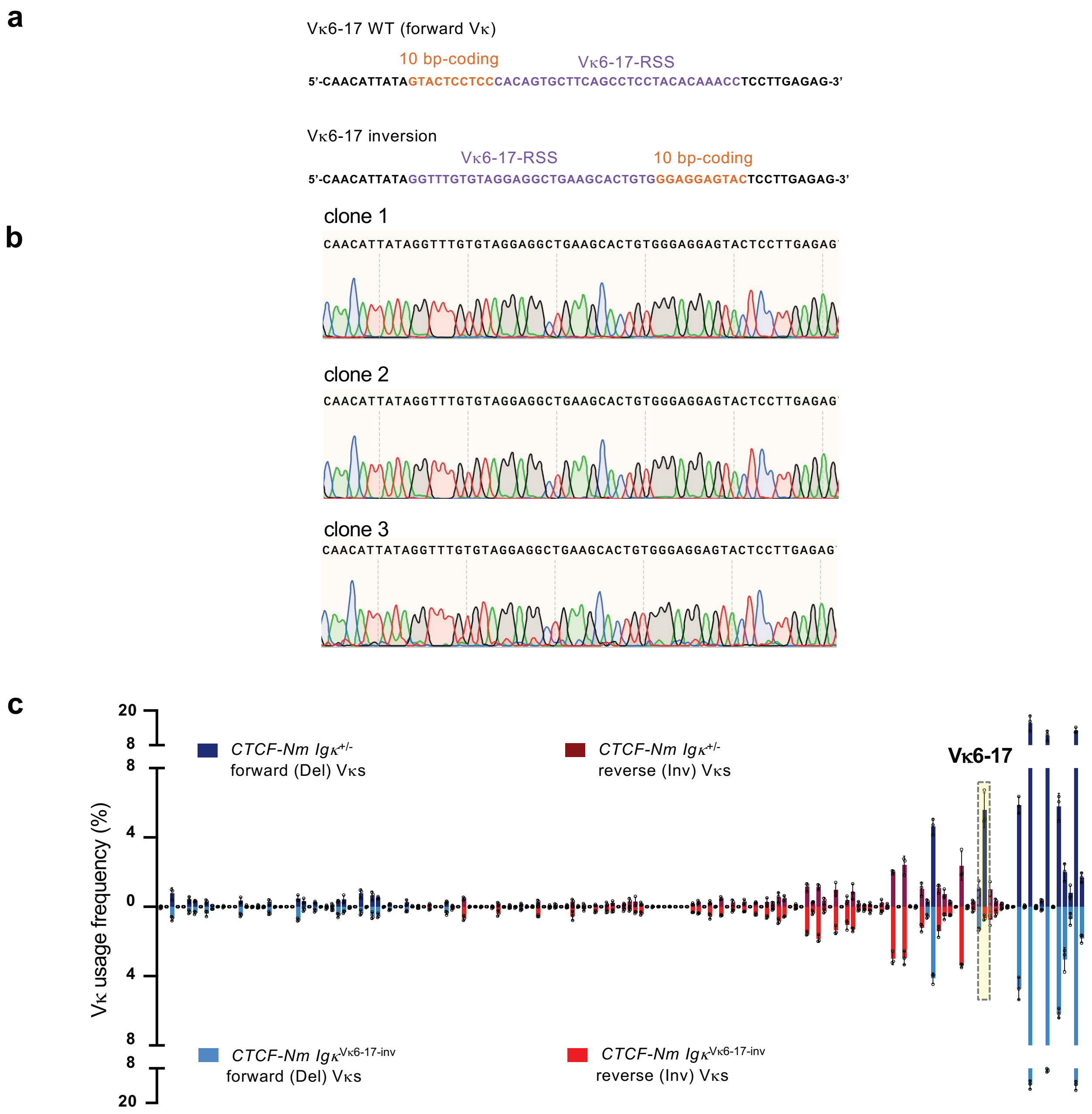

Supplementary Figure 3. Characterization of the *CTCF-Nm Igκ*$^{Vκ6\text{-}17\text{-}inv}$ lines. (**a**) Diagram of the Vκ6-17-inversion mutation. (**b**) Sanger sequencing confirmation of the three biologically independent clones with Vκ6-17-inversion, derived from the *CTCF-Nm Igκ*$^{+/-}$ line. (**c**) HTGTS-V(D)J-Seq analysis of Vκ usage frequency in *CTCF-Nm Igκ*$^{+/-}$ (top) and *CTCF-Nm Igκ*$^{Vκ6\text{-}17\text{-}inv}$ (bottom) lines. Data are shown as mean ± SD from three independent replicates.

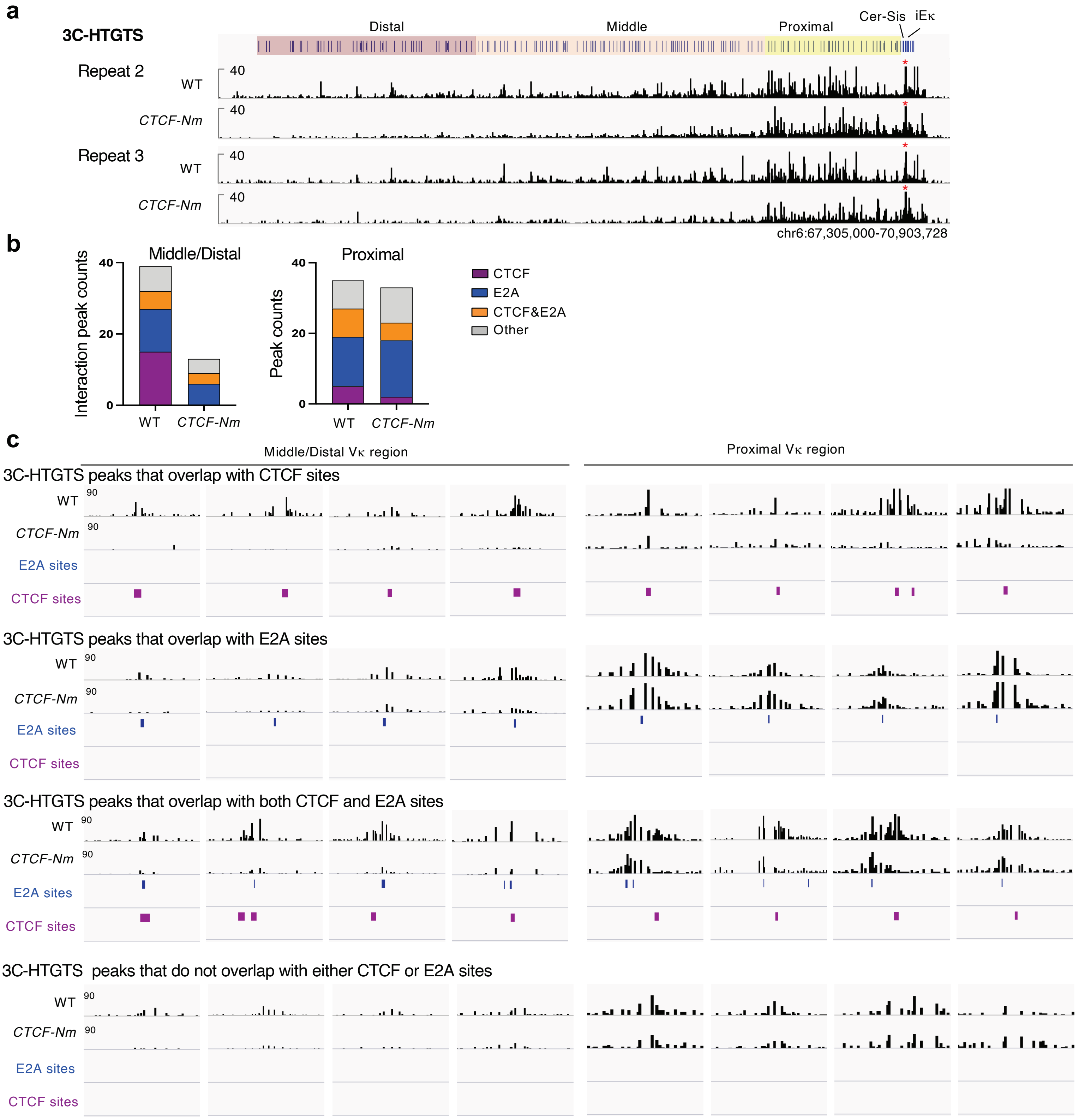

Supplementary Figure 4. Characterization of the impact of the *CTCF-Nm* mutation on *Igκ* chromatin interaction. (**a**) Additional independent 3C-HTGTS replicates (Cer bait) displaying *Igκ* interaction profiles in WT and *CTCF-Nm* cells. Red star marks bait site. (**b**) Analysis of interaction peaks. Bar graphs displaying the counts of Cer-interaction peaks, categorized into four groups: overlap with CTCF sites, overlap with E2A sites, overlap with both CTCF and E2A sites, and others that do not overlap with either CTCF or E2A sites. These groups are shown for the middle/distal and proximal Vκ regions. Only interaction peaks detected in more than one replicate are included for analysis. (**c**) Representative interaction peaks for the four categories described in panel b for WT and *CTCF-Nm* mutant lines. Data are plotted from bedGraphs generated from pooled libraries of three independent replicates. Left: peaks in the middle/distal Vκ region; Right: peaks in the proximal Vκ region.

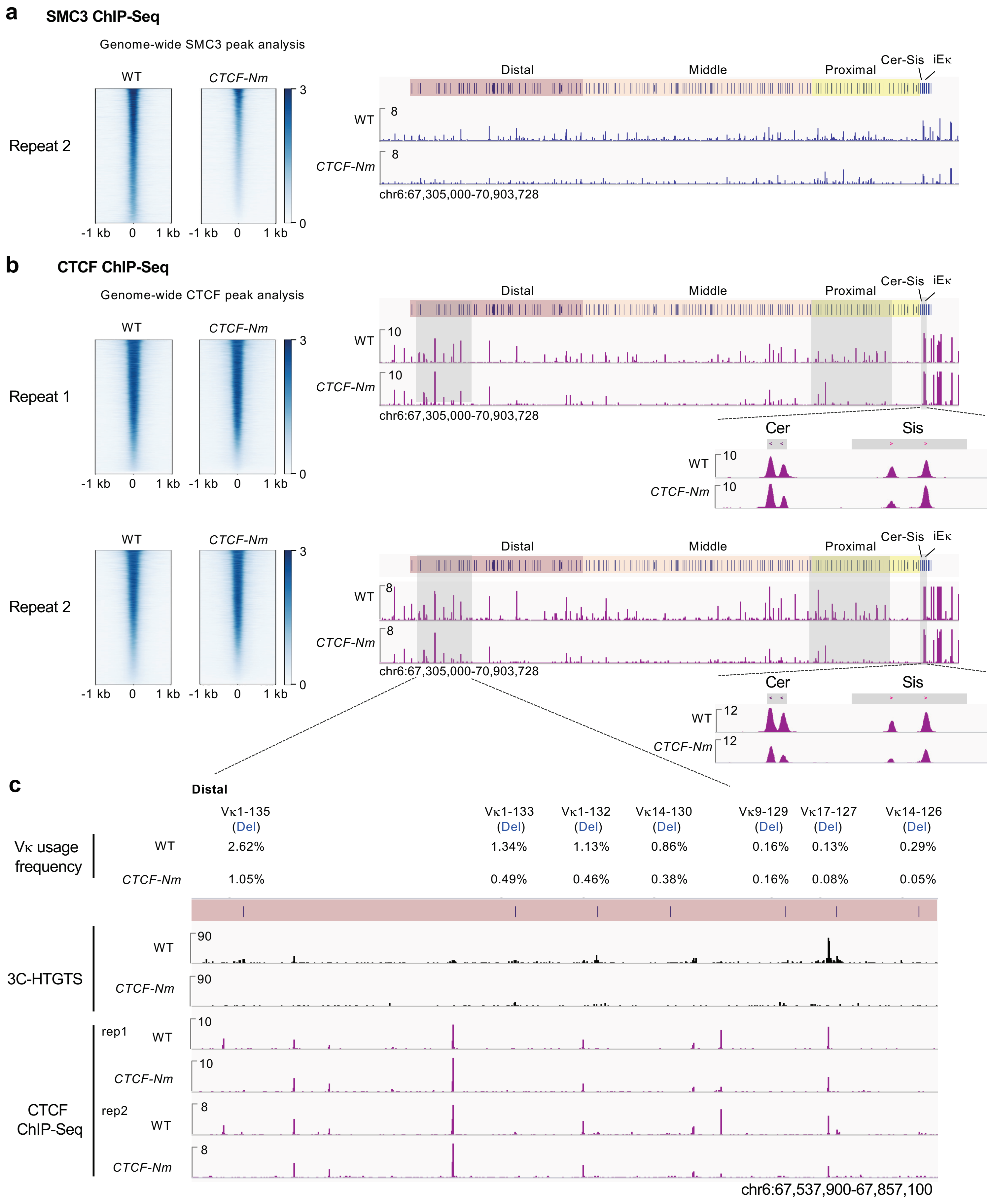

Supplementary Figure 5,

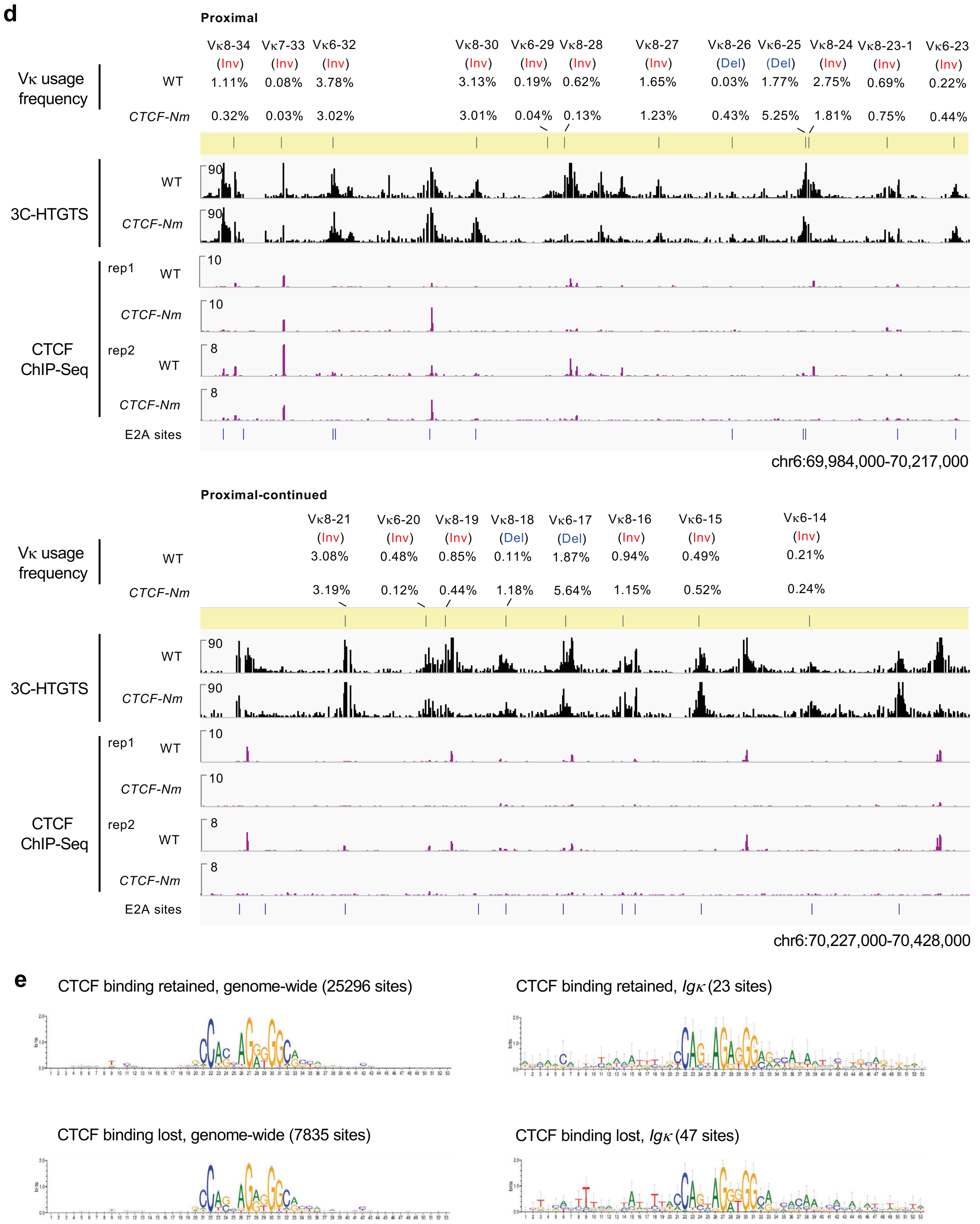

Supplementary Figure 5, continued. See next page for caption.

Supplementary Figure 5. Characterization of the impact of *CTCF-Nm* mutation on chromatin binding of cohesin and CTCF. (**a**) Additional independent SMC3 ChIP-Seq replicates for WT and *CTCF-Nm* lines. Left, Genome-wide SMC3 peak analysis. Right, SMC3 accumulation profiles at the *Igκ* locus. (**b**) CTCF ChIP-Seq analysis for WT and *CTCF-Nm* cells. Left, Genome-wide CTCF peak analysis. Right, CTCF binding profiles across the entire *Igκ* locus, with a magnified view of the Cer-Sis region. (**c**) Enlarged view of a 400-kb distal Vκ region highlighted in panel b. CTCF binding in this region is comparatively less affected by the CTCF-Nm mutation than other Vκ CTCF sites. ChIP-Seq profiles are shown alongside 3C-HTGTS (Cer bait) interaction signals and Vκ usage frequencies. Vκs with a usage frequency > 0.01% are shown. 6 out of 7 Vκs in this region showed significant decrease in usage frequency. (**d**) Enlarged view of a proximal Vκ region highlighted in panel b. (**e**) WebLogos comparing CTCF binding site sequences between WT and *CTCF-Nm* cells, both genome-wide and within the *Igκ* locus. CTCF sites were categorized as either retained or lost in *CTCF-Nm* cells relative to WT. The WebLogos encompassed the 19-bp core motif plus 16 bp upstream and 18 bp downstream sequences. Slight differences in flanking regions are observed for sites in the *Igκ* locus, including (1) elevated frequency of T in flanking positions 3, 9, 18 for the category of CTCF lost, (2) elevated frequency of A in flanking positions 15, 39 and 44 for the category of CTCF lost, (3) elevated frequency of C in flanking positions 6 and 34 for the category of CTCF retained, and (4) elevated frequency of A in flanking positions 36 and 47 for the category of CTCF retained. Statistical significance cannot be robustly estimated due to the limited number of sites.

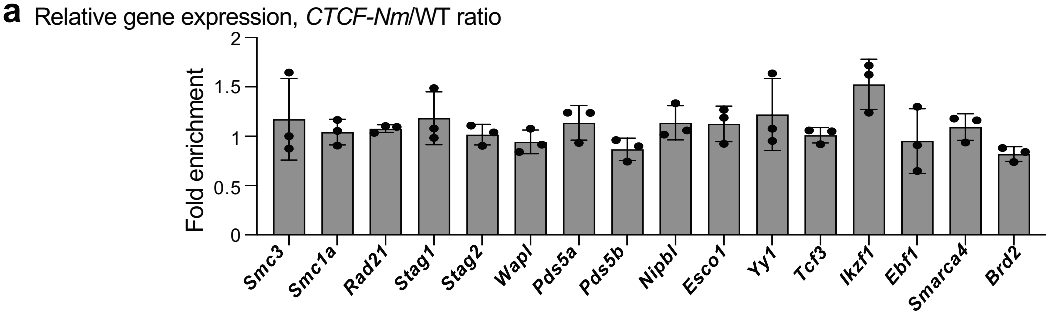

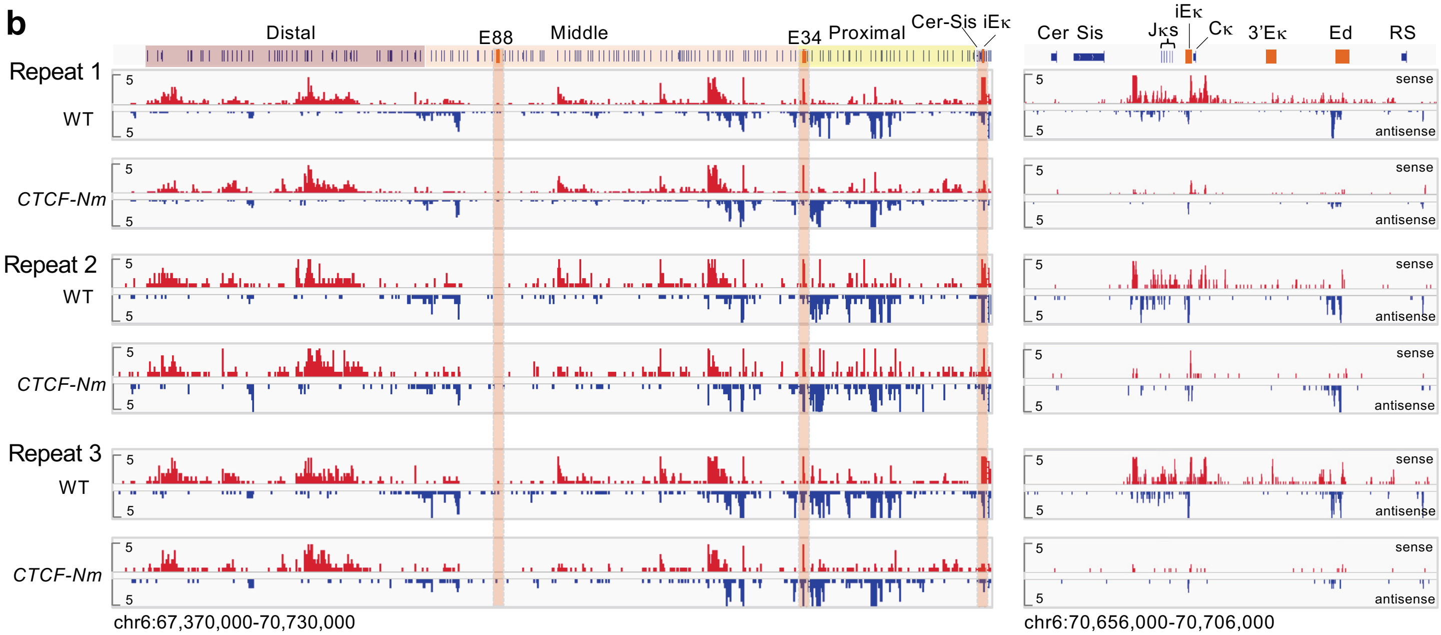

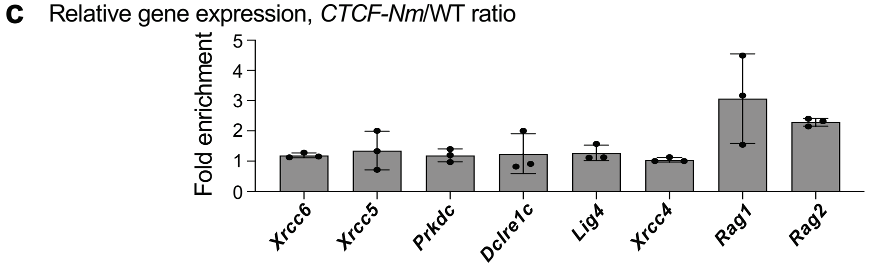

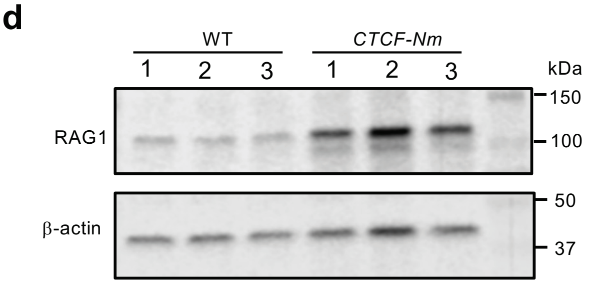

Quantification

	β-Actin	RAG1	RAG1/β-actin ratio	Avg	CTCF-Nm/ WT ratio
WT rep1	1	1	1.00		
WT rep2	1.06	1.05	0.99	1.01	
WT rep3	1.03	1.08	1.05		
CTCF-Nm rep1	1.17	3.3	2.82		3.09
CTCF-Nm rep2	1.7	5.89	3.46	3.13	
CTCF-Nm rep3	1.29	3.99	3.09		

Supplementary Figure 6. Characterization of the impact of the *CTCF-Nm* mutation on gene expression. (**a**) PRO-Seq analysis comparing expression of genes involved in loop extrusion and diffusion between WT and *CTCF-Nm* lines. Ratios of *CTCF-Nm* to WT expression are shown. Data represent mean ± SD from three independent replicates. (**b**) PRO-Seq profiles across the entire *Igκ* locus and a zoomed-in view of the 3' *Igκ* recombination center in WT and *CTCF-Nm* cells. Shown are results from three independent replicates. (**c**) Comparison of expression levels of V(D)J recombination genes in WT and *CTCF-Nm* cells. Ratios of *CTCF-Nm* to WT expression are shown. Data represent mean ± SD from three independent replicates. (**d**) Representative Western blot analysis of RAG1(D708A) expression in WT and three independent *CTCF-Nm* clones. Two independent experiments yielded very similar results (additional repeat is shown in source data). PRO-Seq and Western blotting were performed on RAG1(D708A) cells without WT RAG1 complementation.

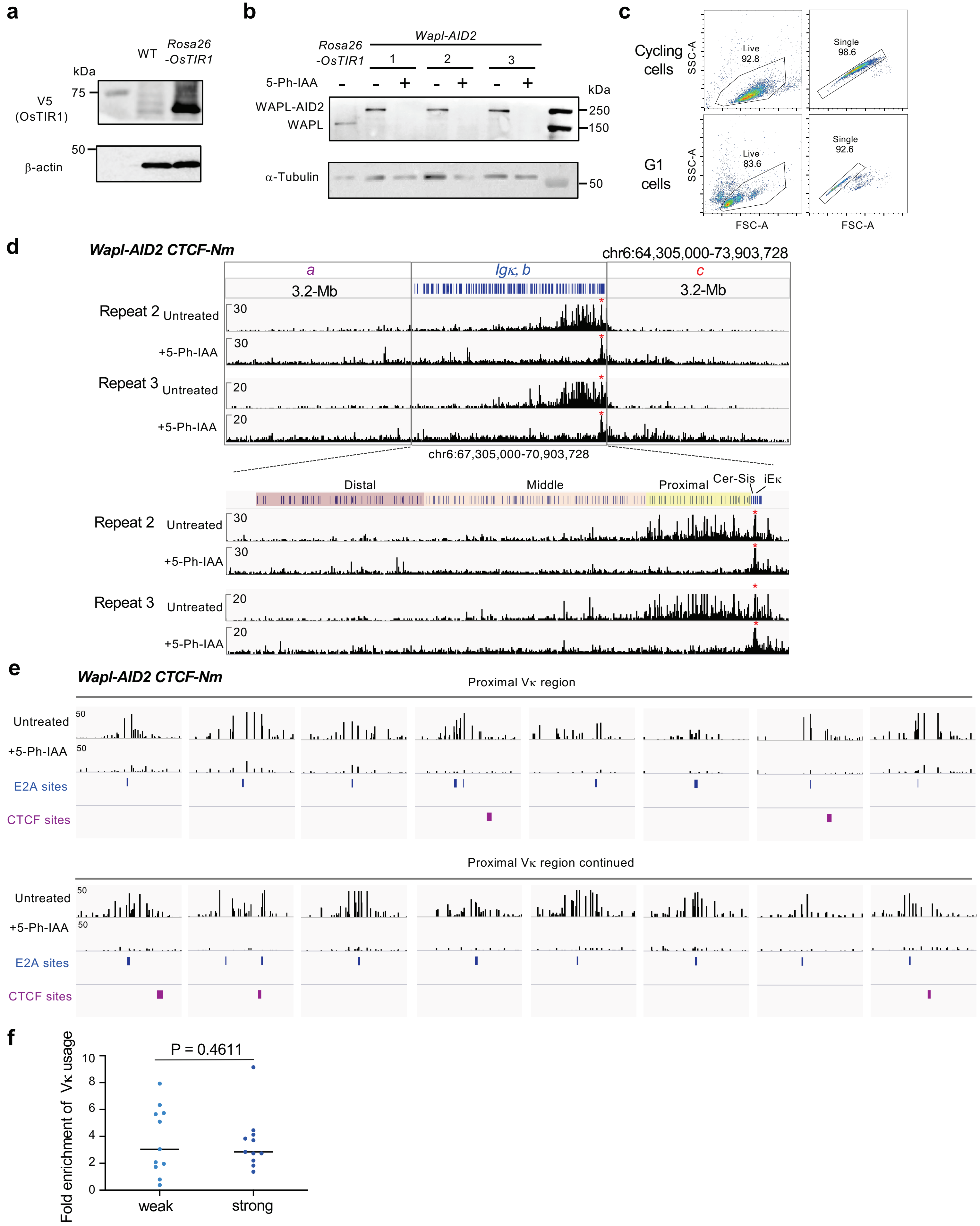

Supplementary Figure 7. see next page for caption.

Supplementary Figure 7. Validation of the WAPL-AID2 system and characterization *of Wapl-AID2 CTCF-Nm* lines via HTGTS-V(D)J-Seq and 3C-HTGTS analysis. (**a**) Western blot confirmation of OsTIR1 expression in the *Rosa26-OsTIR1(F74G)* line. OsTIR1 is tagged with V5 and detected using an anti-V5 antibody. (**b**) Western blot confirmation of efficient WAPL depletion after 4 hours of 5-Ph-IAA treatment in the *Wapl-AID2* line. Data include three biologically independent clones, with the parental *Rosa26-OsTIR1(F74G)* line serving as a control to show the size of wild-type WAPL. (**c**) Flow cytometry gating strategy. The untreated *Wapl-AID2* line is used as an example to show the gating strategy in both cycling and G1-arrested cells. Cells were sequentially gated for live and single cell populations. (**d**) Additional independent 3C-HTGTS replicates (Cer bait) for untreated and 5-Ph-IAA treated *Wapl-AID2 CTCF-Nm* lines, displaying interactions across the region spanning 3.2 Mb upstream to 3.2 Mb downstream of the *Igκ* locus, including a zoomed-in view of interactions at the *Igκ* locus. Red star marks bait site. (**e**) Representative interaction peaks in the proximal Vκ region for untreated and 5-Ph-IAA treated *Wapl-AID2 CTCF-Nm* lines. Data are plotted from bedGraphs generated from pooled libraries of three independent experiments. (**f**) Comparison of fold enrichment for weak and strong distal deletional Vκ RSSs upon WAPL depletion in *CTCF-Nm* cells. The weak group comprises the 11 least-used Vκs (excluding those unused both before and after 5-Ph-IAA treatment), and the strong group comprises the 11 most-used Vκs. Medians for both groups are shown. Each data point shown is mean from three independent replicates. Vκ2-112 is not shown in the figure because it was unused in untreated *Wapl-AID2 CTCF-Nm* cells; however, its usage increased to 0.54% upon WAPL depletion. P value was calculated by the Kolmogorov-Smirnov test.

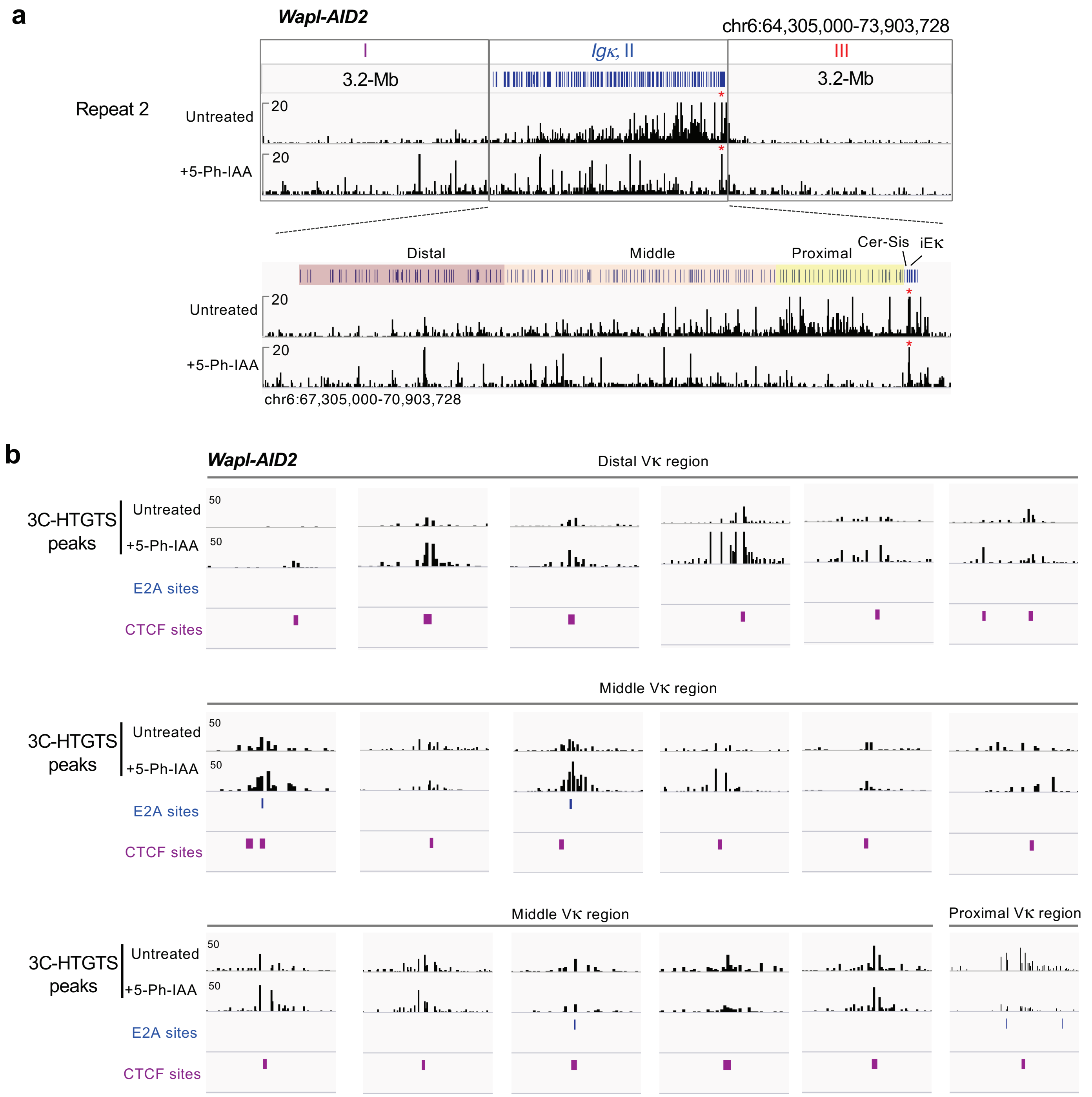

Supplementary Figure 8. Characterization of the *Wapl-AID2* lines. (**a**) Additional 3C-HTGTS replicates (Cer bait) for untreated and 5-Ph-IAA treated *Wapl-AID2* lines. Red star marks bait site. (**b**) Profiles of interaction peaks in the Vκ region for untreated and 5-Ph-IAA treated *Wapl-AID2* lines. Data are plotted from bedGraphs generated from pooled libraries of two independent replicates. An additional 5-Ph-IAA treated *Wapl-AID2* repeat showing very similar pattern was not shown but included in the GSE287935.

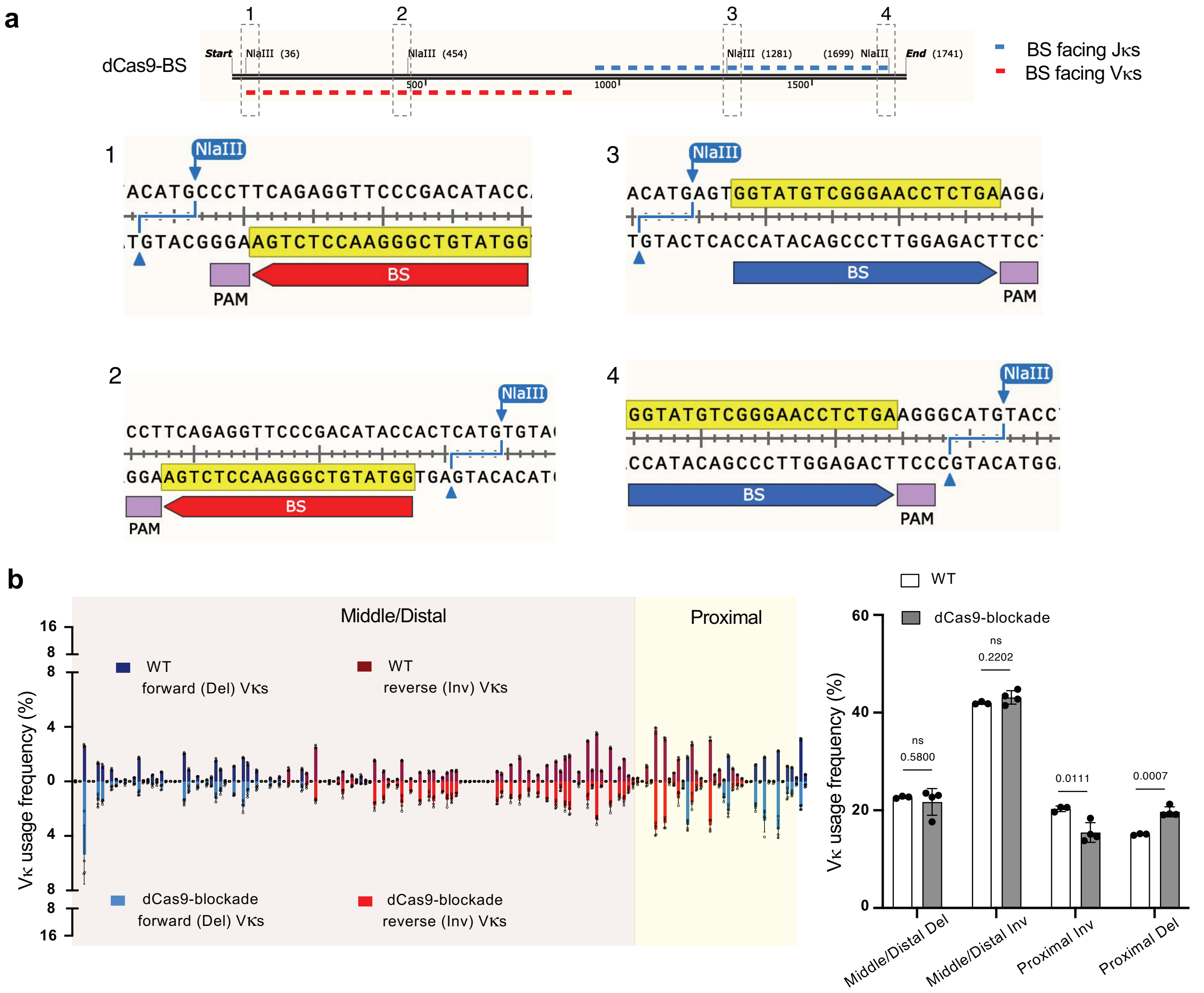

Supplementary Figure 9. Characterization of the dCas9-SunTag blockade system. (**a**) Illustration showing the relative position of the dCas9-SunTag binding sites and NlaIII restriction sites. All 4 NlaIII cleavage sites are located within 5 bp of a BS. (**b**) Left, HTGTS-V(D)J-Seq analysis of Vκ usage frequency (%) in the WT line (top) and the dCas9-SunTag blockade line (bottom). Right, summary of deletional and inversional Vκ usage in proximal versus middle/distal Vκ regions in the WT and dCas9-SunTag blockade lines. Data are shown as mean ± SD from three and four independent repeats, respectively. All P values were calculated using unpaired, two-tailed Student's t test.

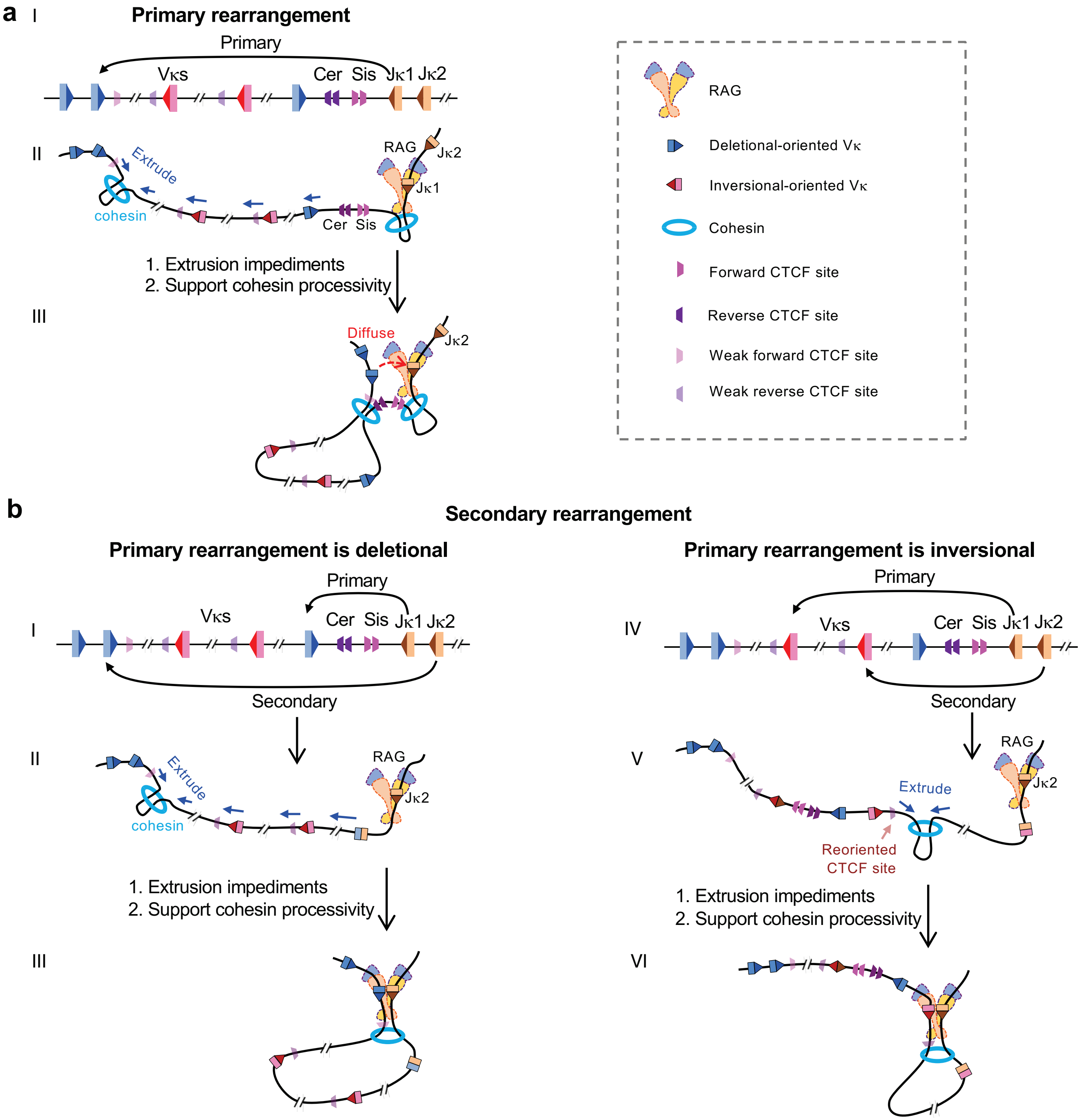

Supplementary Figure 10. Working model of potential roles of CTCF sites within Vκ region during primary and secondary *Igκ* rearrangement. (**a**) Primary rearrangement. An example of a CTCF site promoting forward Vκ usage is shown (I-III). Vκ CTCF sites may act as weak impediments that slow loop extrusion, thereby facilitating the usage of nearby Vκ segments. They may also enhance cohesin processivity to support distal Vκ usage, although this role is likely less critical when the Cer-Sis elements are intact. Cohesin is depicted loading near the Vκ CTCF site, but it may also load anywhere between the CTCF site and the Cer element. (**b**) Secondary rearrangement. Examples are shown for CTCF sites promoting the usage of an originally forward Vκ (I-III) and an originally reverse Vκ (IV-VI). Secondary Vκ-to-Jκ recombination likely occurs predominantly via loop extrusion due to the loss of Cer-Sis elements. Germline CTCF sites in reverse orientation can be inverted by primary inversional joining, converting them into forward CTCF sites that promote nearby Vκ usage during secondary rearrangements. Vκ CTCF sites may similarly serve dual roles, acting as impediments and enhancing cohesin processivity. In the absence of Cer-Sis, their contribution to cohesin processivity may become increasingly important for distal Vκ usage.

Supplementary Table 1. List of DNA oligos used in this study

**Cas9-gRNA oligos (sequences for the targeting strand are shown), ssODNs and PCR validation primers used to generate mutant *v-Abl* pro-B cell lines**		**Source**
gRNA-CTCF(Y226A/F228A) generation	TGATTTTGAGGAAGAACAGC	This paper
ssODN-CTCF(Y226A/F228A)	AAAACCAAAAAGAGCAAACTTCGTTACACAGAAGAGGGCAAAGACGTGGATGTGTCTGTGgccGATgctGAGGAAGAAC<br>AGCAAGAAGGACTGCTGTCTGAGGTTAATGCAGAGAAAGTAGTTGGTAAT	This paper
CTCF(Y226A/F228A) forward, PCR validation	GTCTGTGGCCGATGCTGAGG	This paper
CTCF(Y226A/F228A) reverse, PCR validation	GTACCCACTAGAAGCCTGGC	This paper
CTCF WT forward, PCR validation	AGACGTGGATGTGTCTGTGTA	This paper
CTCF WT reverse, PCR validation	CCTTTACCATGCCGTAGCCC	This paper
gRNA-Igκ deletion-up	GCTGAAGCATGGAAAGGGAC	This paper
gRNA-Igκ deletion-down	GCAGCAGGAAACCATCACCA	This paper
Igκ deletion forward, PCR validation	GCATCACAGAGGAGCTAGCC	This paper
Igκ deletion reverse, PCR validation	AATGTCATTGGCCTGGTCCA	This paper
Igκ WT forward, PCR validation	GCATCACAGAGGAGCTAGCC	This paper
Igκ WT reverse, PCR validation	TCAGCTGGTAGAGTGTGTGC	This paper
gRNA-377 kb-Igκ inversion-up	GCAAACATATCCAACTGCCC	This paper
gRNA-377 kb-Igκ inversion-down	GATAGCAGGAATTTATTTCC	This paper
377 kb-Igκ inversion forward, PCR validation	ACACATGGGTCAGAAGCACC	This paper
377 kb-Igκ inversion reverse, PCR validation	TGTGCCCTTTCGTTTGGAGA	This paper
377 kb-Igκ WT forward, PCR validation	ACACATGGGTCAGAAGCACC	This paper
377 kb-Igκ WT reverse, PCR validation	TCTGAAAGGGCAAAAGACACC	This paper
gRNA-Vκ6-17 inversion with 10 bp CE	AGATGTTTAAACTCTCAAGG	This paper
ssODN-Vκ6-17 inversion with 10 bp CE	ATCTGGGACGGATTTCACTTTCACCATCAGCAGTGTGCAGGCTGAAGACCTGGCAGTTTATTACTGTCAGCAACATTATA<br>GGTTTGTGTAGGAGGCTGAAGCACTGTGGGAGGAGTACTCCTTGAGAGTTTAAACATCTGTGTACACCACACACAGCTCT<br>GGTTATGCACACTTCTCCATTCAGACTTAGAGGTGCCA	This paper
Vκ6-17 inversion forward, PCR validation	GGATTCCACTTTGGACCTGTCG	This paper
Vκ6-17 inversion reverse, PCR validation	CTCTCAAGGAGTACTCCTCCC	This paper
Vκ6-17 WT forward, PCR validation	GGATTCCACTTTGGACCTGTCG	This paper
Vκ6-17 WT reverse, PCR validation	CAGATGTTTAAACTCTCAAGGAGG	This paper
gRNA-Rosa26-OsTIR1 insertion	CAGTCTTTCTAGAAGATGGG	This paper
Rosa26-OsTIR1 insertion forward, PCR validation	GAGACATCCACCTGGAAACCAT	This paper
Rosa26-OsTIR1 insertion reverse, PCR validation	GAGTGAAGCAGAACGTGGGG	This paper
Rosa26-WT forward, PCR validation	GTGCAAGCACGTTTCCGAC	This paper
Rosa26-WT reverse, PCR validation	CATAGTCTAACTCGCGACACTG	This paper
gRNA-Wapl-mAID2 generation	TTACCAAGCACCTGAAGCAA	This paper
Wapl-mAID2 forward, PCR validation	GGGAATATCTTCCAGAAGGAG	This paper
Wapl-mAID2 reverse, PCR validation	CGGTATGATCTCACCGGTG	This paper
Wapl WT forward, PCR validation	GGGAATATCTTCCAGAAGGAG	This paper
Wapl WT reverse, PCR validation	CCCTCTTTAAACCTTGCCTG	This paper
gRNA-dCas9-blockade generation	GCTTTACTTTAACGAGGGAT	This paper
dCas9-binding sites insertion forward, PCR validation	GTGAGTTCGTGACTGCCAAG	This paper
dCas9-binding sites insertion reverse, PCR validation	GCATGTACCTGAGTCGTGACT	This paper
WT (no binding sites) forward, PCR validation	TGACACCGGCCATCACAG	This paper
WT (no binding sites) reverse, PCR validation	GCCACTGAGCCAGCCATC	This paper
**synthetic repetitive gRNA oligo sequence for generation of dCas9 blockade**		
dCas9 binding sites (38 sites)	GAATTCAGTCACGACTCAGGTACATGCCCTTCAGAGGTTCCCGACATACCAGTCACTTGTAATGGCTAACCCTTCAGAG<br>GTTCCCGACATACCCTATAATTTATTGTGTACCCCCTTCAGAGGTTCCCGACATACCAAATCTACTCCCAGTCCCTTCCTT<br>CAGAGGTTCCCGACATACCCATACCAAGAGTCATCCTACCCTTCAGAGGTTCCCGACATACCGAACATTGCTTAACAGG<br>TTACCTTCAGAGGTTCCCGACATACCGATTAACTCCTCCCAGAGAACCTTCAGAGGTTCCCGACATACCTCAATCTTGGA<br>CATTAGGTCCCTTCAGAGGTTCCCGACATACCAGAAAGTAAATAGGTAGTTGCCTTCAGAGGTTCCCGACATACCTCCC<br>ACAGTAGAACAACTCTCCTTCAGAGGTTCCCGACATACCACTCATGTGTAGATGATGTGCCTTCAGAGGTTCCCGACATA<br>CCATCTCCCAGTTCCTATATCTCCTTCAGAGGTTCCCGACATACCCTCCATTACATTCAAACTGACCTTCAGAGGTTCCC<br>GACATACCACAGAACTTTGGGGTTTTGACCTTCAGAGGTTCCCGACATACCCTCTCAACATTGTACAGTTACCTTCAGAG<br>GTTCCCGACATACCCTTAAGACAGGGTTCAGTCCCCTTCAGAGGTTCCCGACATACCTTGTACTTTACTGAAGCAAACCT<br>TCAGAGGTTCCCGACATACCAGCATTATCTACTTTACTGCCCTTCAGAGGTTCCCGACATACCGCTCAACACTAGAGTTT<br>TGCCCTTCAGAGGTTCCCGACATACCGATCATTCTGGCAAACTGACCCTTCAGAGGTTCCCGACATACCCATTGGCAAG<br>CATCGTGGAAGGGACTACACAACCGCAGCCTACTTCCACGATGCTTGCCAATGGGTATGTCGGGAACCTCTGAAGGGTC<br>AGTTTGCCAGAATGATCGGTATGTCGGGAACCTCTGAAGGGCAAAACTCTAGTGTTGAGCGGTATGTCGGGAACCTCTGA<br>AGGGGACTGAACCCTGTCTTAAGGGTATGTCGGGAACCTCTGAAGGTAACTGTACAATGTTGAGAGGGTATGTCGGGAA<br>CCTCTGAAGGTCAAAACCCCAAAGTTCTGTGGTATGTCGGGAACCTCTGAAGGTCAGTTTGAATGTAATGGAGGGTATGT<br>CGGGAACCTCTGAAGGAGATATAGGAACTGGGAGATGGTATGTCGGGAACCTCTGAAGGCACATCATCTACACATGAGT<br>GGTATGTCGGGAACCTCTGAAGGAGAGTTGTTCTACTGTGGGAGGTATGTCGGGAACCTCTGAAGGCAACTACCTATTTA<br>CTTTCTGGTATGTCGGGAACCTCTGAAGGGACCTAATGTCCAAGATTGAGGTATGTCGGGAACCTCTGAAGGTTCTCTGG<br>GAGGAGTTAATCGGTATGTCGGGAACCTCTGAAGGTAACCTGTTAAGCAATGTTCGGTATGTCGGGAACCTCTGAAGGGT<br>AGGATGACTCTTGGTATGGGTATGTCGGGAACCTCTGAAGGAAGGGACTGGGAGTAGATTTGGTATGTCGGGAACCTCTG<br>AAGGGGGTACACAATAAATTATAGGGTATGTCGGGAACCTCTGAAGGGTTAGCCATTACAAGTGACTGGTATGTCGGGA<br>ACCTCTGAAGGGCATGTACCTGAGTCGTGACTGCTAGC	This paper
**RT-PCR primers**		
gRNA1 expression-forward	GGTATGTCGGGAACCTCTGAG	This paper
gRNA1 expression-reverse	CGGTGCCACTTTTTCAAGTT	This paper
Gapdh-forward	TCATCCCTGCATCCACTGGT	This paper
Gapdh-reverse	GCCCTCAGATGCCTGCTTCA	This paper
**ChIP-qPCR primers**		
dCas9 binding sites-forward	GCATGTACCTGAGTCGTGACT	This paper
dCas9 binding sites-reverse	GCCACTGAGCCAGCCATC	This paper
Igκ-RS-forward	CATGTGGGTTTGGCAGACC	This paper

Igκ-RS-reverse	CCTGTCAGCGTAATACAGTGC	This paper
**Southern probe primers**		
Igκ deletion_probe_forward	GGACAGGACAGATGGGCAAA	This paper
Igκ deletion_probe_reverse	CTGCTGCATTCACAGTACACT	This paper
Igκ deletion_probe_nest primer	AGTCCCGCTGTAGGTCTCAT	This paper
377 kb-Igκ inversion-forward	TGCATGTCAACACATCATGGC	This paper
377 kb-Igκ inversion-reverse	GGTGCTTCTGACCCATGTGT	This paper
377 kb-Igκ inversion-nest primer	CATCAACATATTTCAGAGCTGGT	This paper
**HTGTS V(D)J-seq primers**		
Jκ1 bio primer	/5Biosg/TTCCCAGCTTTGCTTACGGAG	This paper
Jκ1 RED (nest) index primer	ACACTCTTTCCCTACACGACGCTCTTCCGATCTNNNNNNAGTGCCAGAATCTGGTTTCAGAG	This paper
BLUE primer	CTCGGCATTCCTGCTGAACCGCTCTTCCGATCTGACTATAGGGCACGCGTGG	Hu et al., 2016 (REF 94)
bridge adapter_top	/5-Phosphorylation/CCACGCGTGCCCTATAGTCGC-NH2	Hu et al., 2016 (REF 94)
bridge adapter_bottom	GCGACTATAGGGCACGCGTGG**NNNNNN**-NH2	Hu et al., 2016 (REF 94)
P5-I5 primer	AATGATACGGCGACCACCGAGATCTACACTCTTTCCCTACACGACGCTCTTCCGATCT	Hu et al., 2016 (REF 94)
P7-I7 primer	CAAGCAGAAGACGGCATACGAGATCGGTCTCGGCATTCCTGCTGAACCGCTCTTC	Hu et al., 2016 (REF 94)
**3C-HTGTS primers**		
Cer bait bio primer	/5Biosg/AATGATCTCACAGTGCCTCC	This paper
Cer bait RED (nest) index primer	ACACTCTTTCCCTACACGACGCTCTTCCGATCTNNNNNNCCAGTTTATCACCAGAGCAAGT	This paper
iEκ bait bio primer	/5Biosg/CCTCTGTATGGCTTCCTTTGGT	This paper
iEκ bait RED (nest) index primer	ACACTCTTTCCCTACACGACGCTCTTCCGATCTNNNNNNCCTTAAGCCAGGGTCTGTATT	This paper
**PRO-Seq primers**		
3' RNA adapter	GAUCGUCGGACUGUAGAACUCUGAAC-/inverted dT/	Mahat et al., 2016 (REF95)
5' RNA adapter	CCUUGGCACCCGAGAAUUCCA	Mahat et al., 2016 (REF95)
RP1	AATGATACGGCGACCACCGAGATCTACACGTTCAGAGTTCTACAGTCCGA	Mahat et al., 2016 (REF95)
RPI-n (index primer)	CAAGCAGAAGACGGCATACGAGATNNNNNNGTGACTGGAGTT CCTTGGCACCCGAGAATTCCA	Mahat et al., 2016 (REF95)

*In ssODN-CTCF(Y226A/F228A) lowercase nucleotides denotes desired mutations.

*In ssODN-CTCF(Y226A/F228A) underlined nucleotides: changed to disrupt the PAM for Cas9-gRNA recognition after successful generation of mutation via SSODN-mediated CRISPR-Cas9 approach, generate a silent mutation.

*In dCas9 binding sites (38 sites) underlined nucleotide denotes dCas9 binding sites (including PAM motif), first 20 orientated toward the Vk region, second 18 orientated toward the Jκ region.

*In index primers, underlined NNNNNN represent the index sequence, 2 to 8 nucloetides in length, unique to each sample in a pooled library.

*In the bridge adapter_bottom, the bolded NNNNNN denotes a machine-generated mixture of six nucleotides with random sequences.

Supplementary Table 2. Antibodies used in this study.

Antibodies	Source	Catalog number	Dilutions or amount used in experiments
Anti-SMC3	Fortis Life Sciences	A300-060A	2.5 ug per ChIP experiment, 5 ug per Co-IP
IgG isotype control	Invitrogen	02-6102	5 ug per Co-IP experiment
Anti-CTCF	Millipore Sigma	07-729	2.5 ug per ChIP experiment
Anti-Cas9	Diagenode	C15310258	1: 1000 for western blotting
Anti-beta-Actin	Cell Signaling Technology	3700T	1: 1000 for western blotting
Anti-RAG1	Abcam	AB172637-1001	1: 1000 for western blotting
Anti-V5	Invitrogen	R960-25	1: 1000 for western blotting
Anti-WAPL	Invitrogen	PA5-38024	1: 500 for western blotting
Anti-alpha-Tubulin	Santa Cruz	sc-23948	1: 1000 for western blotting
Goat anti-Rabbit, secondary	Invitrogen	A27036	1: 10,000 for western blotting
Sheep anti-Mouse, secondary	Cytiva	NA931V	1: 10,000 for western blotting
