# Supplementary material for: CTCF couples long-range loop extrusion and diffusion to mediate a diverse Igκ repertoire
Source: Nat Commun. 2025 Dec 11;17:751. doi: 10.1038/s41467-025-67438-5 (PMC12820099; doi:10.1038/s41467-025-67438-5)
Supplement: Supplementary file 2 — Reporting Summary [file 41467_2025_67438_MOESM2_ESM.pdf]

nature portfolio

Corresponding author(s): Yu Zhang

Last updated by author(s): Nov 20, 2025

Reporting Summary

Nature Portfolio wishes to improve the reproducibility of the work that we publish. This form provides structure for consistency and transparency in reporting. For further information on Nature Portfolio policies, see our Editorial Policies and the Editorial Policy Checklist.

Statistics

For all statistical analyses, confirm that the following items are present in the figure legend, table legend, main text, or Methods section.

n/a	Confirmed	
☐	☒	The exact sample size (*n*) for each experimental group/condition, given as a discrete number and unit of measurement
☐	☒	A statement on whether measurements were taken from distinct samples or whether the same sample was measured repeatedly
☐	☒	The statistical test(s) used AND whether they are one- or two-sided<br>*Only common tests should be described solely by name; describe more complex techniques in the Methods section.*
☒	☐	A description of all covariates tested
☐	☒	A description of any assumptions or corrections, such as tests of normality and adjustment for multiple comparisons
☐	☒	A full description of the statistical parameters including central tendency (e.g. means) or other basic estimates (e.g. regression coefficient) AND variation (e.g. standard deviation) or associated estimates of uncertainty (e.g. confidence intervals)
☐	☒	For null hypothesis testing, the test statistic (e.g. *F*, *t*, *r*) with confidence intervals, effect sizes, degrees of freedom and *P* value noted<br>*Give P values as exact values whenever suitable.*
☒	☐	For Bayesian analysis, information on the choice of priors and Markov chain Monte Carlo settings
☒	☐	For hierarchical and complex designs, identification of the appropriate level for tests and full reporting of outcomes
☒	☐	Estimates of effect sizes (e.g. Cohen's *d*, Pearson's *r*), indicating how they were calculated

Our web collection on statistics for biologists contains articles on many of the points above.

Software and code

Policy information about availability of computer code

Data collection	Next generation sequencing data were collected via Illumina sequencing platforms (MiSeq or NovaSeq 6000). Data generated from MiSeq and NovaSeq 6000 were demultiplexed viaTranslocPreprocess.pl, a published pipeline available at (http://robinmeyers.github.io/transloc_pipeline/).
Data analysis	HTGTS V(D)J-seq and 3C-HTGTS data were processed through a published pipeline available at (http://robinmeyers.github.io/transloc_pipeline/). 3C-HTGTS peak calling was performed via the published pipeline available at (https://github.com/Yyx2626/HTGTS_related/tree/main/3CHTGTS_related). ChIP-Seq libraries were aligned to mm9 or modified genomes using Bowtie2, processed using samtools v1.8, deduplicated using Picard (https://broadinstitute.github.io/picard/) and normalized using bamCoverage with the --CPM flag. CTCF and E2A peak sites were called by MACS2 narrow peak calling function. For genome-wide peak analysis, reference peak annotation was generated using MACS2 peak calling function. Heatplots were generated using deepTools2. Protein identification for IP-qMS was performed using MaxQuant 1.5.5.1. Statistical analysis in all assays were performed via GraphPad Prism9. PRO-Seq libraries were were aligned to the mm9 genome, and duplicate reads were removed using samtools markdup. Libraries were strand-separated based on alignment orientation and normalized to reads per million. Gene expression was quantified using the HTSeq tool. HTGTS-V(D)J-Seq analysis were profiled using GraphPad Prism9. 3C-HTGTS, ChIP-Seq and PRO-Seq analysis were profiled using IGV 2.6.2.

For manuscripts utilizing custom algorithms or software that are central to the research but not yet described in published literature, software must be made available to editors and reviewers. We strongly encourage code deposition in a community repository (e.g. GitHub). See the Nature Portfolio guidelines for submitting code & software for further information.

Data

Policy information about availability of data

All manuscripts must include a data availability statement. This statement should provide the following information, where applicable:

- Accession codes, unique identifiers, or web links for publicly available datasets
- A description of any restrictions on data availability
- For clinical datasets or third party data, please ensure that the statement adheres to our policy

High-throughput sequencing data generated in this study have been deposited at the Gene Expression Omnibus (GEO) database. HTGTS-V(D)J-Seq data is under accession code GSE287940 [https://www.ncbi.nlm.nih.gov/geo/query/acc.cgi?acc=GSE287940]. 3C-HTGTS data is under accession code GSE287937 [https://www.ncbi.nlm.nih.gov/geo/query/acc.cgi?acc=GSE287937]. ChIP-Seq data is under accession code GSE287935 [https://www.ncbi.nlm.nih.gov/geo/query/acc.cgi?acc=GSE287935]. PRO-Seq data is under accession code GSE305325 [https://www.ncbi.nlm.nih.gov/geo/query/acc.cgi?acc= GSE305325]. Mass spectrometry data generated in this study have been deposited at the Proteomics Identification (PRIDE) database under accession code PXD058006 [https://www.ebi.ac.uk/pride/archive/projects/PXD058006]. E2A ChIP-Seq was extracted from a previously published GEO accession code GSM546523 [https://www.ncbi.nlm.nih.gov/geo/query/acc.cgi?acc=GSM546523]

Research involving human participants, their data, or biological material

Policy information about studies with human participants or human data. See also policy information about sex, gender (identity/presentation), and sexual orientation and race, ethnicity and racism.

Reporting on sex and gender	*Use the terms sex (biological attribute) and gender (shaped by social and cultural circumstances) carefully in order to avoid confusing both terms. Indicate if findings apply to only one sex or gender; describe whether sex and gender were considered in study design; whether sex and/or gender was determined based on self-reporting or assigned and methods used.*<br>*Provide in the source data disaggregated sex and gender data, where this information has been collected, and if consent has been obtained for sharing of individual-level data; provide overall numbers in this Reporting Summary. Please state if this information has not been collected.*<br>*Report sex- and gender-based analyses where performed, justify reasons for lack of sex- and gender-based analysis.*
Reporting on race, ethnicity, or other socially relevant groupings	*Please specify the socially constructed or socially relevant categorization variable(s) used in your manuscript and explain why they were used. Please note that such variables should not be used as proxies for other socially constructed/relevant variables (for example, race or ethnicity should not be used as a proxy for socioeconomic status).*<br>*Provide clear definitions of the relevant terms used, how they were provided (by the participants/respondents, the researchers, or third parties), and the method(s) used to classify people into the different categories (e.g. self-report, census or administrative data, social media data, etc.)*<br>*Please provide details about how you controlled for confounding variables in your analyses.*
Population characteristics	*Describe the covariate-relevant population characteristics of the human research participants (e.g. age, genotypic information, past and current diagnosis and treatment categories). If you filled out the behavioural & social sciences study design questions and have nothing to add here, write "See above."*
Recruitment	*Describe how participants were recruited. Outline any potential self-selection bias or other biases that may be present and how these are likely to impact results.*
Ethics oversight	*Identify the organization(s) that approved the study protocol.*

Note that full information on the approval of the study protocol must also be provided in the manuscript.

Field-specific reporting

Please select the one below that is the best fit for your research. If you are not sure, read the appropriate sections before making your selection.

- [x] Life sciences
- [ ] Behavioural & social sciences
- [ ] Ecological, evolutionary & environmental sciences

For a reference copy of the document with all sections, see nature.com/documents/nr-reporting-summary-flat.pdf

Life sciences study design

All studies must disclose on these points even when the disclosure is negative.

Sample size	No statistical methods were used to predetermine sample size for all experiments. Sample sizes were chosen based on previous studies in this field that used similar sample sizes to generate reproducible results.
Data exclusions	No data were excluded from analysis.
Replication	All experiments were performed with at least two independent biological replicates.
Randomization	Experiments were not randomized. Each experiment was performed with identified controls and mutant strains. Randomization was not relevant to the study as the study does not involve participant groups.

Blinding	Investigators were not blinded to allocation during experiments and outcome assessment. Blinding was not possible as investigators need to verify the control and matched mutant strains before each experiment. Also, based on previous studies in this field, these assays do not require blinding.

Reporting for specific materials, systems and methods

We require information from authors about some types of materials, experimental systems and methods used in many studies. Here, indicate whether each material, system or method listed is relevant to your study. If you are not sure if a list item applies to your research, read the appropriate section before selecting a response.

Materials & experimental systems

n/a	Involved in the study	
☐	☒	Antibodies
☐	☒	Eukaryotic cell lines
☒	☐	Palaeontology and archaeology
☒	☐	Animals and other organisms
☒	☐	Clinical data
☒	☐	Dual use research of concern
☒	☐	Plants

Methods

n/a	Involved in the study	
☐	☒	ChIP-seq
☐	☒	Flow cytometry
☒	☐	MRI-based neuroimaging

Antibodies

Antibodies used

Anti-WAPL antibody: Invitrogen, Cat# PA5-38024, Lot# V13076867.
Anti-V5 antibody Invitrogen, Cat# R960-25, Clone SV5-Pk1; Lot# 2245071.
Anti-beta-Actin antibody Cell Signaling Technology, Cat# 3700T, Clone 8H10D10.
Anti-beta-Tubulin antibody Santa Cruz, Cat# sc-23948, Clone B-5-1-2, Lot# F0719.
Anti-Cas9-antibody Diagenode, Cat# C15310258, Lot# A2508-004.
Anti-SMC3 antibody Fortis Life Sciences, Cat# A300-060A, Lot# 11.
Anti-CTCF antibody Sigma-Aldrich, Cat# 07-729, Lot# 3275487.
Anti-RAG1 antibody Abcam, Cat# AB172637, Clone EPRAGR1, Lot# 1040760-16.
Goat Anti-Rabbit secondary antibody Invitrogen, Cat# A27036
Sheep Anti-Mouse secondary antibody Cytiva, Cat# NA931V

Validation

Goat Anti-Rabbit secondary antibody (Invitrogen, A27036), manufacturer's validation: https://www.thermofisher.com/antibody/product/Goat-anti-Rabbit-IgG-Heavy-chain-Secondary-Antibody-Recombinant-Superclonal/A27036
This antibody was validated for western blot in publications including:
Rittenhouse NL, Gohil R, Arricastres JE, Dowen JM. Unraveling the cohesin-chromatin interface: identifying protein interactions that modulate chromosome structure and function. Epigenetics Chromatin. 2025 Jun 2;18(1):31. doi: 10.1186/s13072-025-00596-4.

Sheep Anti-Mouse secondary antibody (Cytiva NA931V), manufacturer's validation: https://www.cytivalifesciences.com/en/us/products/items/amersham-ecl-hrp-conjugated-antibodies-p-06260
This antibody was validated for western blotting in publications including:
Grande RC, Lin CC, Cammer M, Emesom ED, Khurram MA, Boutell C, Denes LT, Lionnet T, Wilson AC, Mohr I. De novo assembly of RNA m6A modification factors into viral genome-associated nuclear bodies drives HCMV RNA accumulation. Cell Rep. 2025 Jul 22;44(7):115826. doi: 10.1016/j.celrep.2025.115826.

Cas9 antibody (Diagenode, C15310258), manufacturer's validation: https://www.diagenode.com/en/p/crispr-cas9-polyclonalantibody
This antibody was validated for ChIP in the following paper:
Howe, FS. et al. CRISPRi is not strand-specific at all loci and redefines the transcriptional landscape. Elife. Oct 23;6. pii: e29878. doi: 10.7554/eLife.29878 (2017).

beta-Actin antibody (Cell Signaling Technology, 3700S), manufacturer's validation: https://www.cellsignal.com/products/primaryantibodies/b-actin-8h10d10-mouse-mab/3700
This antibody was validated for western blotting in publications including:
Lahrouchi, N. et al., Homozygous frameshift mutations in FAT1 cause a syndrome characterized by colobomatousmicrophthalmia, ptosis, nephropathy and syndactyly. Nat Commun. Mar 12;10(1):1180. doi: 10.1038/s41467-019-08547-w. (2019)

Wapl antibody (Invitrogen, PA5-38024), manufacturer's validation: https://www.thermofisher.com/antibody/product/WAPL-Antibody-Polyclonal/PA5-38024
This antibody was validated for western blotting in publications including:
Dai HQ, Hu H, Lou J, et al. Loop extrusion mediates physiological Igh locus contraction for RAG scanning. Nature. 2021;590(7845):338-343. doi:10.1038/s41586-020-03121-7

V5 antibody (Invitrogen, SV5-Pk1), manufacturer's validation:
https://www.thermofisher.com/antibody/product/V5-Tag-Antibody-clone-SV5-Pk1-Monoclonal/R960-25

This antibody was validated for western blotting in publications including:
Gouveia Roque C, Chung KM, McCurdy EP, et al. CREB3L2-ATF4 heterodimerization defines a transcriptional hub of Alzheimer's disease gene expression linked to neuropathology. Sci Adv. 2023;9(9):eadd2671. doi:10.1126/sciadv.add2671

alpha-Tubulin antibody (Santa Cruz Biotechnology, B-5-1-2), manufacturer's validation:
https://www.scbt.com/p/alpha-tubulin-antibody-b-5-1-2?srsltid=AfmBOoozDt_uH-sgFcdUMs36rKU8mtnGLCafpVlxzYV1ZN8ihhdaUqLT
This antibody was validated for western blotting in publications including:
Tapia Del Fierro A, den Hamer B, Benetti N, et al. SMCHD1 has separable roles in chromatin architecture and gene silencing that could be targeted in disease. Nat Commun. 2023;14(1):5466. doi:10.1038/s41467-023-40992-6

SMC3 antibody (Fortis Life Sciences, A300-060A), manufacturer's validation:
https://www.fortislife.com/products/primary-antibodies/rabbit-anti-smc3-antibody/BETHYL-A300-060
This antibody was validated for ChIP-Seq in publications including:
Ladurner R, Kreidl E, Ivanov MP, et al. Sororin actively maintains sister chromatid cohesion. EMBO J. 2016;35(6):635-653. doi:10.15252/embj.201592532

CTCF antibody (Sigma Aldrich, 07-729):
https://www.sigmaaldrich.com/US/en/product/mm/07729
This antibody was validated for ChIP-Seq in publications including:
Martin D, Pantoja C, Fernández Miñán A, et al. Genome-wide CTCF distribution in vertebrates defines equivalent sites that aid the identification of disease-associated genes. Nat Struct Mol Biol. 2011;18(6):708-714. doi:10.1038/nsmb.2059

RAG1 antibody (Abcam, AB172637):
https://www.abcam.com/en-us/products/primary-antibodies/rag1-antibody-epragr1-ab172637
This antibody was validated for Western blotting in publications including:
Chen C, Chen B, Wang Y, et al. Sarco/endoplasmic reticulum Ca2+-ATPase (SERCA) activity is required for V(D)J recombination. J Exp Med (2021) 218 (8): e20201708. https://doi.org/10.1084/jem.20201708

Eukaryotic cell lines

Policy information about cell lines and Sex and Gender in Research

Cell line source(s)	The RAG1(D708A), Emu-Bcl2+ mouse v-Abl pro-B cell line is a gift from Dr. David Schatz. This line is a male cell line and was used as the parental line to generate mutant derivatives including the CTCF-Nm line and the Wapl-AID2 line. The CTCF-Nm line was used to generate the CTCF-Nm Igk+/- line, which was further used to generate the CTCF-Nm Igk-377-inv and the CTCF-Nm Igk-Vk6-17-inv lines. The Wapl-AID2 line was used to generate the Wapl-AID2 CTCF-Nm line, which was further used to generate the BS-only and dCas9-blockade lines.
Authentication	The RAG1(D708A), Emu-Bcl2+ mouse v-Abl pro-B cell line was validated by PCR. The CTCF-Nm line was validated by PCR and sequencing, the Wapl-AID2 line was validated by PCR, western blot and flow cytometry analysis. The CTCF-Nm Igk+/- and CTCF-Nm Igk-377-inv lines were validated by PCR and southern blot analysis. The CTCF-Nm Igk-Vk6-17-inv and the Wapl-AID2 CTCF-Nm lines were validated by PCR and sequencing. The BS-only and dCas9-blockade lines were validated by PCR, sequencing, western blot, RT-qPCR and ChIP-qPCR.
Mycoplasma contamination	The cell lines were not tested for mycoplasma contamination.
Commonly misidentified lines (See ICLAC register)	No commonly misidentified cell lines were used.

Plants

Seed stocks	*Report on the source of all seed stocks or other plant material used. If applicable, state the seed stock centre and catalogue number. If plant specimens were collected from the field, describe the collection location, date and sampling procedures.*
Novel plant genotypes	*Describe the methods by which all novel plant genotypes were produced. This includes those generated by transgenic approaches, gene editing, chemical/radiation-based mutagenesis and hybridization. For transgenic lines, describe the transformation method, the number of independent lines analyzed and the generation upon which experiments were performed. For gene-edited lines, describe the editor used, the endogenous sequence targeted for editing, the targeting guide RNA sequence (if applicable) and how the editor was applied.*
Authentication	*Describe any authentication procedures for each seed stock used or novel genotype generated. Describe any experiments used to assess the effect of a mutation and, where applicable, how potential secondary effects (e.g. second site T-DNA insertions, mosiacism, off-target gene editing) were examined.*

ChIP-seq

Data deposition

- [x] Confirm that both raw and final processed data have been deposited in a public database such as GEO.
- [x] Confirm that you have deposited or provided access to graph files (e.g. BED files) for the called peaks.

Data access links<br>*May remain private before publication.*	GEO accession GSE287935: https://www.ncbi.nlm.nih.gov/geo/query/acc.cgi?acc=GSE287935
Files in database submission	IP_SMC3_WT_rep1.bw<br>IP_SMC3_WT_rep2.bw<br>IP_SMC3_CTCF-Nm_rep1.bw<br>IP_SMC3_CTCF-Nm_rep2.bw<br>IP_CTCF_WT_rep1.bw<br>IP_CTCF_WT_rep2.bw<br>IP_CTCF_CTCF-Nm_rep1.bw<br>IP_CTCF_CTCF-Nm_rep2.bw<br>IP_SMC3_WT_rep1_R1.fq.gz<br>IP_SMC3_WT_rep1_R2.fq.gz<br>IP_SMC3_WT_rep2_R1.fq.gz<br>IP_SMC3_WT_rep2_R2.fq.gz<br>IP_SMC3_CTCF-Nm_rep1_R1.fq.gz<br>IP_SMC3_CTCF-Nm_rep1_R2.fq.gz<br>IP_SMC3_CTCF-Nm_rep2_R1.fq.gz<br>IP_SMC3_CTCF-Nm_rep2_R2.fq.gz<br>IP_CTCF_WT_rep1_R1.fq.gz<br>IP_CTCF_WT_rep1_R2.fq.gz<br>IP_CTCF_WT_rep2_R1.fq.gz<br>IP_CTCF_WT_rep2_R2.fq.gz<br>IP_CTCF_CTCF-Nm_rep1_R1.fq.gz<br>IP_CTCF_CTCF-Nm_rep1_R2.fq.gz<br>IP_CTCF_CTCF-Nm_rep2_R1.fq.gz<br>IP_CTCF_CTCF-Nm_rep2_R2.fq.gz<br>Input_WT_rep1_R1.fq.gz<br>Input_WT_rep1_R2.fq.gz<br>Input_WT_rep2_R1.fq.gz<br>Input_WT_rep2_R2.fq.gz<br>Input_CTCF-Nm_rep1_R1.fq.gz<br>Input_CTCF-Nm_rep1_R2.fq.gz<br>Input_CTCF-Nm_rep2_R1.fq.gz<br>Input_CTCF-Nm_rep2_R2.fq.gz
Genome browser session<br>(e.g. UCSC)	No longer applicable.

Methodology

Replicates	Two biologically independent experiments were performed for each genotype analyzed.
Sequencing depth	All libraries are sequenced using paired-end 150 bp reads.<br>IP_SMC3_WT_rep1: 15533878 reads, 54.09% uniquely mapped reads<br>IP_SMC3_WT_rep2: 14175868 reads, 54.64% uniquely mapped reads<br>IP_SMC3_CTCF-Nm_rep1: 15283617 reads, 44.91% uniquely mapped reads<br>IP_SMC3_CTCF-Nm_rep2: 17286102 reads, 50.83% uniquely mapped reads<br>IP_CTCF_WT_rep1: 12590478 reads, 47.51% uniquely mapped reads<br>IP_CTCF_WT_rep2: 27199079 reads, 51.21% uniquely mapped reads<br>IP_CTCF_CTCF-Nm_rep1: 12402106 reads, 48.64% uniquely mapped reads<br>IP_CTCF_CTCF-Nm_rep2: 16263054 reads, 44.90% uniquely mapped reads
Antibodies	Anti-SMC3 antibody Fortis Life Sciences Cat# A300-060A; lot# 11.<br>Anti-CTCF antibody Sigma-Aldrich Cat# 07-729; lot# 3275487.
Peak calling parameters	We used bowtie2 for read mapping: bowtie2 -x bowtie2_indexes -1 READS_R1.fastq -2 READS_R2.fastq -p 6 --non-deterministic -S OUTPUT_DIR/SAMPLE_NAME.sam<br>We use MAC2 to call narrow peaks, use command macs2 callpeak -t "$IP_bam_file" -c "$Input_bam_file" -f BAM -g mm -n "$folder_name" --outdir "$output_dir/$folder_name" --SPMR --verbose 0

Data quality	We relied on MACS2 algorithm to detect significant peaks, obtaining 6-27 thousand narrow peaks at FRD5% and above 5 fold enrichment for SMC3 ChIP-Seq libraries and obtaining 25-29 thousand narrow peaks at FRD5% and above 5 fold enrichment for CTCF ChIP-Seq libraries.
Software	ChIP-Seq were aligned to mm9 or modified genomes using Bowtie2, processed using samtools v1.8, deduplicated using Picard (https://broadinstitute.github.io/picard/) and normalized using bamCoverage with the --CPM flag. CTCF and E2A peak sites were called by MACS2 narrow peak calling function. For genome-wide peak analysis, reference peak annotation was generated using MACS2 peak calling function. Heatplots were generated using deepTools2.

Flow Cytometry

Plots

Confirm that:

- [x] The axis labels state the marker and fluorochrome used (e.g. CD4-FITC).
- [x] The axis scales are clearly visible. Include numbers along axes only for bottom left plot of group (a 'group' is an analysis of identical markers).
- [ ] All plots are contour plots with outliers or pseudocolor plots.
- [ ] A numerical value for number of cells or percentage (with statistics) is provided.

Methodology

Sample preparation	Rosa26-OsTIR1 and untreated and 1 uM 5-Ph-IAA treated Wapl-AID2 v-Abl pro-B cells were collected at indicated time points and cell cycle stages, spun down, PBS washed once, and resuspended in PBS with 2.5% FBS prior for flow cytometry analysis.
Instrument	Attune CytPix
Software	FlowJo v10.10
Cell population abundance	To assess Wapl degradation efficiency, plots are normalized to 10,000 cells for each genotype and then all live cells are gated for analysis (75-90%).
Gating strategy	All live cells were gated for analysis, followed by single cell gating, histogram was then plotted to show the shift of tdTomato intensity before and after 5-Ph-IAA treatment for the Wapl-AID2 line and in comparison to the parental control strain that does not express tdTomato.

- [x] Tick this box to confirm that a figure exemplifying the gating strategy is provided in the Supplementary Information.
